# Opiates and Anæsthetics

**Published:** 1888-07-14

**Authors:** 


### OPIATES AND ANÆSTHETICS.

What is the difference between an anæsthetic and a narcotic? An anæsthetic is an agent capable of preventing pain. The word is derived from the same root as "æsthetic," which denotes unusual perception or sensation, the prefix "a" or "an" denoting absence, and hence anæsthetic means something causing absence of sensation. An opiate or narcotic may also reduce pain, but it induces sleep in addition, though not profound enough to prevent the patient from feeling acute pain, nor to render him unconscious when undergoing an operation. Narcotics exercise a stupefying influence over the intellect, while small doses of anæsthet cs may paralyse sensation—that is, prevent the patient feeling pain—yet leave his reasoning powers clear, as the following cases show.

A patient was to undergo an operation for removal of a tooth, and it was agreed that he was to take nitrous-oxide gas, and as long as he had any sensation left was to move his finger. However, he did not like inhaling too much gas, and to free himself from it ceased to move his finger, and the operation began. Afterwards, the patient explained that though he was conscious the whole time of each stage of the operation, he felt no pain. Under amylene an amputation was performed, and the patient, though conscious, felt no pain, and declared afterwards that when the surgeon began to saw the bone he doubted whether the operation had begun.

Pain as a symptom of disease must not be regarded as the whole disease, though a physician uses pain as a guide to the real nature of the ailment. Pain is often in inverse relation to the extent or seriousness of the disease. Thus a man may have a decayed tooth, which will cause him excruciating pain for months, whilst another man may have his liver riddled with abcesses or cancerous growths, or part of his brain destroyed, or his lungs in an advanced state of disease, and be conscious of no pain. In fact, in advanced lung disease there is a remarkable joyousness and brightness, and the patient will often say: "Relieve me of my cough, and I shall be quite well." It is often asked if the progress of medical science in curing and preventing disease is equal to that displayed in its diagnosis? The medical man will reply that by devoting much attention to disease itself, to the study of its real nature, causes and consequences, he can do most for its relief; but by merely mitigating pain—which is only a symptom of the disease, not the disease itself—by the application of a remedy, the curative process may even be stopped.

Pain is often of service, and its departure an element of danger. It is useful as showing the existence of an evil, and if relieving pain makes us forget that we must still apply remedies for the disease, it leads us far wrong. Pain shows the existence of something unnatural and abnormal, and often makes us conscious of organs of which in health we are unaware. For instance, as long as a man's sight is good he not conscious of his eyes; but as age advances, and his eyes become dim, he is painfully conscious of the defect, and reading becomes difficult. If he applied cocaine, or some other opiate, his relief would only be temporary, and it is much better for him to take the warning given by pain by going to an oculist and getting glasses. Sometimes a patient is conscious of an organ, not only through pain, but from a feeling of discomfort and restlessness. Take, for instance, the case of a man who had a tumour on the brain causing epileptic fits, and had part of his brain removed. No defect of intellectual power resulted, but he had a feeling of something wrong or left out, and this sensation was almost worse than the pain he had previously endured. This condition may also be brought about by paying special concentration of attention to any part, as in hysteria or monomania, etc. The same condition may also be brought about by lack of sleep and rest, and such sufferers, instead of being treated with indifference, call for specially gentle and sympathetic treatment and help, often more than those actually suffering from some organic disease.

There are many anæsthetics, and it is necessary to consider the reasons for choosing one rather than another. Chloroform produces effects more rapidly and pleasantly, and with fewer disagreeable after-effects than ether, while anæsthesia is caused in three or four minutes with the former, and in from five to twenty minutes with the latter. Another drawback to ether is that the patient becomes so saturated with it that it emanates from him for many hours afterwards, whereas this is not the case with chloroform, which has been given with great success and safety. Ten years ago it was considered safer than ether, but as the average number of deaths from chloroform has been higher than from ether, the latter is at present considered the safer of the two. Various kinds of apparatus have been invented for the administration of chloroform, some of which are rather elaborate, but usually a cone of flannel is quite sufficient. During inhalation there is very often great excitement, and sometimes even screaming, as though the patient were suffering severely, whilst in reality he is quite unconscious, and only the subject of spasmodic action. In the lower animals the nervous system is not developed as it is with us, and is not capable of governing movement, whilst the higher up the scale we advance the greater do we find the power of adapting movements to requirements. In this respect a man under the influence of an anæsthetic resembles the lower animals.

